# Contrasting nickel and zinc hyperaccumulation in subspecies of *Dichapetalum gelonioides* from Southeast Asia

**DOI:** 10.1038/s41598-018-26859-7

**Published:** 2018-06-25

**Authors:** Philip Nti Nkrumah, Guillaume Echevarria, Peter D. Erskine, Antony van der Ent

**Affiliations:** 1 Centre for Mined Land Rehabilitation, Sustainable Minerals Institute, The University of Queensland, Queensland, Australia; 2 Université de Lorraine, INRA, Laboratoire Sols et Environnement, Nancy, 54000 France

## Abstract

Hyperaccumulator plants have the unique ability to concentrate specific elements in their shoot in concentrations that can be thousands of times greater than in normal plants. Whereas all known zinc hyperaccumulator plants are facultative hyperaccumulators with only populations on metalliferous soils hyperaccumulating zinc (except for *Arabidopsis halleri* and *Noccaea* species that hyperaccumulate zinc irrespective of the substrate), the present study discovered that *Dichapetalum gelonioides* is the only (zinc) hyperaccumulator known to occur exclusively on ‘normal’ soils, while hyperaccumulating zinc. We recorded remarkable foliar zinc concentrations (10 730 µg g^−1^, dry weight) in *Dichapetalum gelonioides* subsp. *sumatranum* growing on ‘normal’ soils with total soil zinc concentrations of only 20 µg g^−1^. The discovery of zinc hyperaccumulation in this tropical woody plant, especially the extreme zinc concentrations in phloem and phloem-fed tissues (reaching up to 8465 µg g^−1^), has possible implications for advancing zinc biofortification in Southeast Asia. Furthermore, we report exceptionally high foliar nickel concentrations in *D*. subsp. *tuberculatum* (30 260 µg g^−1^) and >10 wt% nickel in the ash, which can be exploited for agromining. The unusual nickel and zinc accumulation behaviour suggest that *Dichapetalum*-species may be an attractive model to study hyperaccumulation and hypertolerance of these elements in tropical hyperaccumulator plants.

## Introduction

Hyperaccumulators are plants that accumulate exceptional concentrations of certain trace elements in their above-ground biomass when growing in their natural habitats^1–3^. At present, the hyperaccumulation threshold values are set at: 100 µg g^−1^ for Cd, Se and Tl; 300 µg g^−1^ for Co, Cr and Cu; 1000 µg g^−1^ for As, Ni and Pb; 3000 µg g^−1^ for Zn; and 10 000 µg g^−1^ for Mn^3^. These unique plants have attracted growing attention from science, mainly because of their prospects for agromining (*e.g*. cultivating high biomass hyperaccumulator plants and recovering valuable products from the harvested biomass)^4–8^, and similarly for potential use in the phytoremediation of polluted sites^9–13^. More recently, hyperaccumulators, especially for Zn, have attracted considerable interest for the insights that they may yield for biofortification of food crops for human consumption^14,15^. Current research in the field of hyperaccumulator plants aims to provide insights into the physiological and biomolecular mechanisms underlying the hyperaccumulation trait, and the ecological significance and function, as well as potential applications^16–22^.

More than 700 hyperaccumulator plants have been discovered from across the globe, the majority (>70%) of which hyperaccumulate Ni^3^. Most Ni hyperaccumulator plants are from the Brassicaceae-family originating from Europe and from the Phyllanthaceae-family originating from tropical regions^2,23,24^. Nickel hyperaccumulator plant species occur on ultramafic outcrops, with some species being ‘obligate’ (*i.e*. restricted to that substrate and always hyperaccumulating Ni), and other species are able to grow on a range of different soils, with only ultramafic populations hyperaccumulating nickel^25,26^. In comparison to Ni hyperaccumulator plants, only a small number of Zn hyperaccumulator plant species (15 taxa) are known to date^27^; about 60% of which are in the Brassicaceae family. Zinc hyperaccumulator species include the herbaceous plants *Arabidopsis halleri*, *Arabis paniculata*, *Noccaea caerulescens*, *Noccaea praecox, Noccaea fendleri* (Brassicaceae), *Picris divaricata* (Asteraceae), *Potentilla griffithii* (Rosaceae) and *Sedum plumbizincicola* (Crassulaceae)^28–34^. Among these species, *A. halleri* and *N. caerulescens* are key model species for Ni and Zn-Cd hyperaccumulation, and the two most intensively studied hyperaccumulator species globally^35–44^. *Arabidopsis halleri* and *N*. *caerulescens* can accumulate extremely high concentrations of Cd-Zn (up to 53 450 µg g^−1^ Zn and 2890 µg g^−1^ Cd) and/or Ni (16 200 µg g^−1^) when growing on Zn-Pb-Cd-enriched metalliferous soils or on Ni-Co-Mn-enriched ultramafic soils^39–42^. However, Zn hyperaccumulation is a species-wide trait in these species^29,43^, and these two species are also able to hyperaccumulate Zn when growing on ‘normal’ soils with only background concentrations of Zn (*i.e.* soils with 1.00–300 µg g^−1^ total Zn)^41,44^. Reeves *et al*.^41^ analysed field collected specimens of *N. caerulescens* and the corresponding ‘normal’ soils in France and Luxembourg, and reported foliar Zn concentrations of 3230–8890 µg g^−1^ when occurring on soils with Zn concentrations of only 115–274 µg g^−1^.

The family Dichapetalaceae has three genera: *Dichapetalum*, *Stephanopodium* and *Tapura*^45^. The largest genus is *Dichapetalum* with >150 species^46^ occurring predominantly in tropical and subtropical regions, with most species distributed in Africa^45^. *Dichapetalum gelonioides* occurs throughout Southeast Asia, and has five subspecies: *D*. subsp. *andamanicum*, *D*. subsp. *gelonioides*, *D*. subsp. *pilosum*, *D*. subsp. *sumatranum* and *D*. subsp. *tuberculatum*^46,47^. Apart from *D*. subsp. *andamanicum*, all the other subspecies occur in Sabah (Malaysian Borneo), in primary lowland mixed Dipterocarp forest. All of the *Dichapetalum*-species are scandent scrubs, frequently climbing when small, but growing to small trees (<6 m in height with a <20 cm diameter at breast height (DBH) bole) when mature. Whereas *D*. subsp. *tuberculatum* and subsp. *sumatranum* have glabrous leaves, *D*. subsp. *pilosum* has pubescent (hairy) leaves. The key taxonomic characteristic is the fruits, which are either three-lobed and smooth (subsp. *tuberculatum*) or two-lobed and hairy (subsp. *sumatranum*).

Nearly three decades ago, Baker *et al*.^46^ discovered that *D. gelonioides* subsp. *tuberculatum* from the Philippines (on Palawan Island) hyperaccumulated Ni when growing on ultramafic soils. This then led to a survey of herbarium specimens of other *Dichapetalum gelonioides* subspecies from Southeast Asia. The analysis of herbarium specimen fragments confirmed that *D*. subsp. *tuberculatum* is a strong Ni hyperaccumulator when growing on ultramafic soils with up to 26 650 µg g^−1^ foliar Ni, and a strong Zn hyperaccumulator when growing on normal soils with up to 30 000 µg g^−1^ foliar Zn^46^. *Dichapetalum* subsp*. sumatranum* and *D*. subsp*. pilosum* are strong Zn hyperaccumulators when growing on normal soils, reaching up to 15 660 µg g^−1^ and 26 360 µg g^−1^ foliar Zn, respectively^46^. The discovery of Zn hyperaccumulation was unexpected, and unexplained at the time, and even though an important finding, it did not lead to further investigations. Baker *et al*.^46^ concluded that exceptional Zn accumulation in the *D. gelonioides* subsp. *tuberculatum* specimen from Sumatra (which had 30 000 µg g^−1^ foliar Zn) must have been due to unknown base metal mineralisation at that site, because of concomitant elevated foliar Cd (9 µg g^−1^), Pb (88 µg g^−1^) and Co (200 µg g^−1^) concentrations in this specimen. The phytochemistry of Ni in *D. gelonioides* subsp. *tuberculatum* was studied by Homer *et al*.^48^ and revealed that the aqueous plant extracts contain 18% Ni, 24% citric acid and 43% malic acid. Recently, systematic screening of herbarium specimens by our team using handheld X-ray Fluorescence Spectroscopy (XRF) including all specimens (91) in the genus *Dichapetalum* held at the Forest Research Centre Herbarium in Sepilok, Sabah, Malaysia (see Supplementary Table 1) revealed that:

- *Dichapetalum gelonioides* subsp. *tuberculatum* ﻿is a strong Ni hyperaccumulator ﻿with up to 31 700 µg g^−^^1^ Ni
- *Dichapetalum gelonioides* subsp. *tuberculatum* can also attain high Zn concentrations of up to 3990 µg g^−1^
- *Dichapetalum gelonioides* subsp. *pilosum* is a strong Zn hyperaccumulator with up to 10 600 µg g^−1^ Zn
- *Dichapetalum gelonioides* subsp. *sumatranum* is also a strong Zn hyperaccumulator with up to 10 500 µg g^−1^ Zn
- *Dichapetalum grandifolium* has elevated Zn concentrations of up to 1300 µg g^−1^

Herbarium specimens were also measured with handheld XRF at the Queensland Herbarium for several Asia-Pacific species of *Dichapetalum*, including *D. papuanum*, *D. timoriense*, *D. tricapsulare* and *D. sessiliflorum*, none of which hyperaccumulated Ni or Zn. However, a specimen of *D. vitiense* from Fiji (collected in 1947 at Vanua Levu, on the summit ridge of Mt Numbuiloa, east of Lambasa, 500–590 m asl in forest) was confirmed as a Zn hyperaccumulator with 7800 μg g^−1^ foliar Zn. This finding raises the prospect that *Dichapetalum* species other than *D. gelonioides* could be Zn hyperaccumulators.

*Dichapetalum gelonioides* is a tropical species that occurs throughout Southeast Asia and attains high biomass, thereby overcoming the main drawbacks that limit the use of *A. halleri* and *N*. *caerulescens* for biofortification in tropical areas. Apart from the biofortification potential, *D. gelonioides* may be useful to advance our knowledge on hyperaccumulation of trace elements, particularly Zn. All Zn hyperaccumulator plant species known to date are facultative hyperaccumulators, and hence the majority occur primarily on normal soils (where they do not hyperaccumulate Zn), and have some populations on metalliferous soils where they do hyperaccumulate Zn^41^. Therefore, these Zn hyperaccumulators behave essentially as ‘Indicators’ (*sensu* Baker^49^) in that the level of accumulation in the shoot is strongly dependant on soil Zn concentrations. The main exceptions are *A. halleri* and *N*. *caerulescens* that have populations on normal soils that are also able to hyperaccumulate Zn^41^. *Dichapetalum gelonioides* is the only (Zn) hyperaccumulator known to occur exclusively on normal soils, while hyperaccumulating Zn. As such, *Dichapetalum gelonioides* provides an attractive model to study Zn tolerance, uptake, translocation, accumulation and detoxification, and may ultimately be useful for applications in the field of biofortification. This study aims to decipher the extent and variability of Ni and Zn hyperaccumulation traits in wild *D. gelonioides* subspecies occurring in Sabah.

## Results

### Elemental concentrations in different parts of *Dichapetalum gelonioides* plants

**Figure 1:**
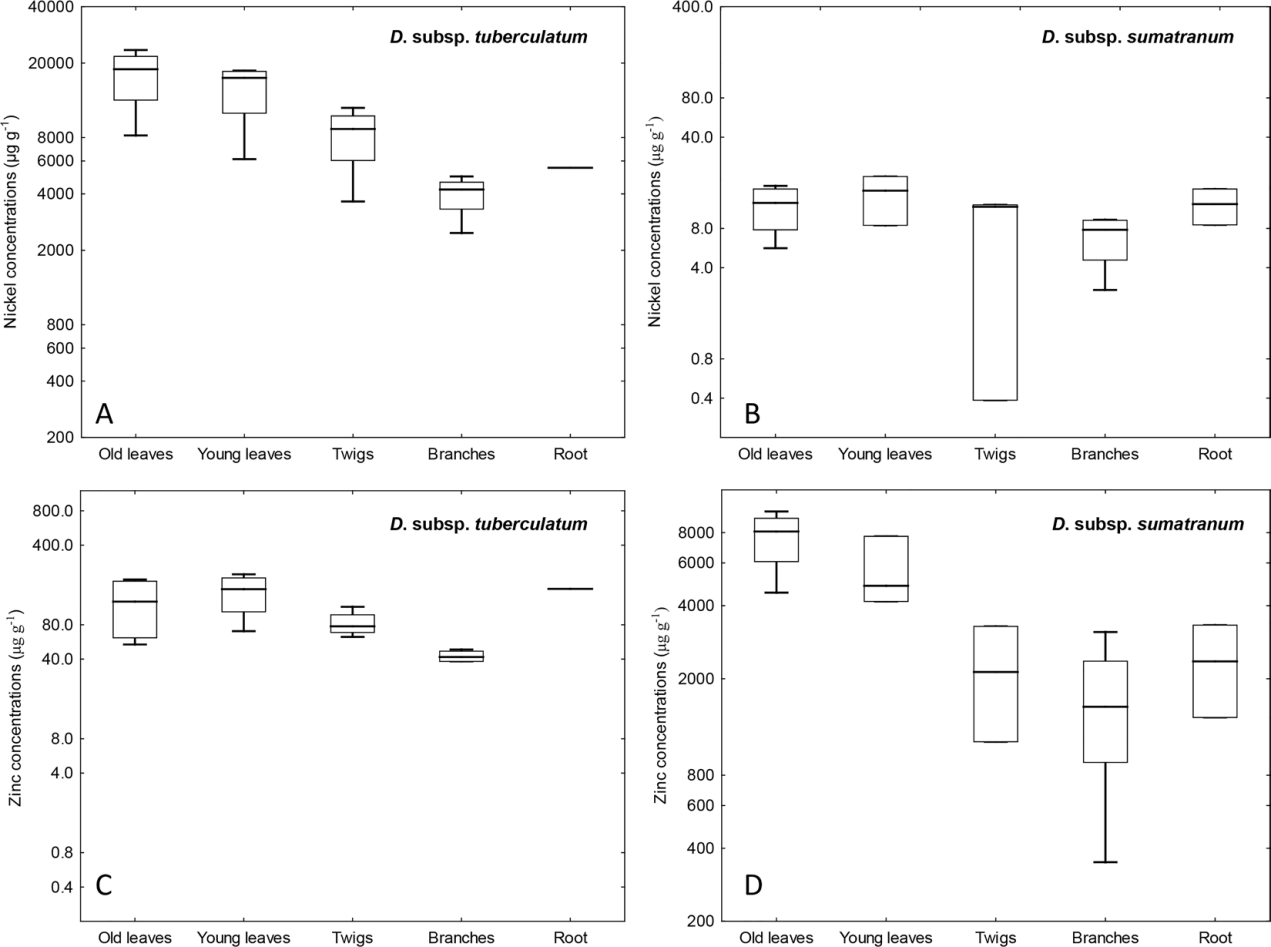
Differences in the accumulation of Ni and Zn in *Dichapetalum*-species: (**A**) Nickel concentrations in *D*. subsp. *tuberculatum*, (**B**) Nickel concentrations in *D*. subsp. *sumatranum*, (**C**) Zinc concentrations in *D*. subsp. *tuberculatum*, and (**D**) Zinc concentrations in *D*. subsp. *sumatranum*.

The elemental concentrations in the different plants parts of the various *Dichapetalum gelonioides* subspecies clearly show that *D. gelonioides* subsp. *tuberculatum* hyperaccumulates Ni, whereas *D*. subsp. *pilosum* and *D*. subsp. *sumatranum* are Zn hyperaccumulators (Table 1). The Ni concentrations in the leaves of *D*. subsp. *tuberculatum* are remarkable (the mean concentration is >1wt%), in contrast to that of *D*. subsp. *pilosum* and *D*. subsp. *sumatranum*, which have low Ni concentrations (mean is 10 and 15 µg g^−1^ respectively) (Fig. 1). On the contrary, the Zn concentrations are substantially elevated in the leaves of *D*. subsp. *pilosum* and *D*. subsp. *sumatranum* (>3000 µg g^−1^), but that of the *D*. subsp. *tuberculatum* are below 300 µg g^−1^ (Fig. 1). The concentrations of the major elements, Ca, K, Mg and S are relatively elevated (mean concentrations >1000 µg g^−1^), whereas the concentrations of the trace elements Co and Fe are uniformly low (mean < 50 µg g^−1^) in the leaves of all subspecies. Whereas the concentrations of Mn are elevated in the leaves of *D*. subsp. *pilosum* (>500 µg g^−1^), that of *D*. subsp. *tuberculatum* are low (mean <50 µg g^−1^). The highest Ni and Zn concentrations are recorded in the old leaves of *D*. subsp. *tuberculatum* (23 480 µg g^−1^) and *D*. subsp. *sumatranum* (9780 µg g^−1^) respectively, followed by young leaves with up to 18 230 µg g^−1^ for *D*. subsp. *tuberculatum* and 7720 µg g^−1^ for *D*. subsp. *sumatranum* respectively. The Ni concentrations in the branches and twigs of *D*. subsp. *tuberculatum* are substantially enriched (>1000 µg g^−1^). For *D*. subsp. *pilosum* and *D*. subsp. *sumatranum*, the Zn concentrations in the branches and twigs are elevated, but the values are below 3000 µg g^−1^. Unripe and matured fruits of *D*. subsp. *sumatranum* are also high in Zn (1955 and 2785 µg g^−1^ respectively). The bark/phloem of *D*. subsp. *sumatranum* has exceptionally high Zn concentrations (8465 µg g^−1^), whereas that of *D*. subsp. *pilosum* is elevated in Zn but below 3000 µg g^−1^. Whilst the roots of *D*. subsp. *tuberculatum* have high Ni concentrations (5510 µg g^−1^), those of *D*. subsp. *pilosum* and *D*. subsp. *sumatranum* are enriched in Zn (770 and 1380–3325 µg g^−1^ respectively). In *Dichapetalum* subsp. *tuberculatum*, the mean concentration of Ni in the old leaves (17 190 µg g^−1^) far exceed that in the roots (5510 µg g^−1^). Likewise, for *D*. subsp. *pilosum* and *D*. subsp. *sumatranum*, the Zn concentrations in the old leaves (6600 and 7620 µg g^−1^ respectively) are higher than in the roots (770 and 2355 µg g^−1^ respectively).

**Table 1:** Bulk elemental concentrations in different plant tissues in *Dichapetalum gelonioides* subsp. *tuberculatum*, subsp. *sumatranum* and subsp. *pilosum* (values are given in ranges and means in µg g^−1^).

*Dichapetalum gelonioides* subsp.	n	Ni	Zn	Al	Ca	Co	Fe	K	Mg	Mn	P	S
**Young leaves**												
subsp. *tuberculatum*	4	6135–18230 [14420]	70–220 [155]	10–25 [20]	2350–4510 [3030]	10–15 [10]	20–35 [25]	3070–5920 [4905]	1460–5370 [3060]	20–40 [30]	375–770 [490]	865–2300 [1690]
subsp. *sumatranum*	3	8.5–20 [15]	4160–7720 [5570]	10–15 [15]	4635–5590 [5110]	8.0–10 [9.3]	20–25 [20]	12650–16770 [14190]	1610–1820 [1735]	35–240 [160]	605–715 [655]	3740–7150 [5785]
subsp. *pilosum*	1	10	4090	55	5085	5.0	35	13305	3320	895	575	4235
**Old Leaves**												
subsp. *tuberculatum*	4	8210–23480 [17190]	55–200 [125]	12–57 [30]	2660–6420 [4110]	10–15 [10]	20–60 [45]	805–2495 [1470]	1700–5780 [3300]	25–70 [40]	245–460 [380]	1165–2810 [2180]
subsp. *sumatranum*	4	5.5–15 [10]	4525–9780 [7620]	23–81 [40]	5570–11070 [8235]	8.0–15 [10]	25–60 [40]	6090–11815 [8760]	1345–1880 [1730]	60–295 [205]	430–565 [505]	5035–7890 [6840]
subsp. *pilosum*	1	5.0	6600	100	5415	10	45	6945	1770	1085	570	2765
**Branches**												
subsp. *tuberculatum*	4	2470–4960 [3965]	35–50 [45]	4.5–10 [7.0]	290–945 [525]	9.0–10 [9.5]	8.0–15 [10]	530–1080 [830]	365–1525 [685]	5.5–15 [9.5]	165–375 [265]	255–395 [325]
subsp. *sumatranum*	4	2.5–9.5 [6.5]	350–3115 [1635]	6.0–25 [15]	985–2855 [1745]	9.4–10 [10]	6.7–25 [15]	1987–5380 [3825]	107–445 [275]	12–50 [25]	135–555 [310]	600–1745 [1195]
subsp. *pilosum*	1	5.0	495	25	1090	5.0	15	3095	490	50	285	765
**Twigs**												
subsp. *tuberculatum*	4	3645–11520 [8235]	65–115 [85]	9.2–25 [15]	1015–1785 [1305]	7.5–10 [9.5]	15–25 [20]	1230–3355 [1910]	1025–3570 [1705]	8.5–25 [15]	250–925 [520]	425–600 [515]
subsp. *sumatranum*	4	0.4–10 [8.1]	1100–3285 [2170]	10–60 [30]	1965–2800 [2515]	7.6–8.6 [8.2]	10–40 [25]	6335–6400 [6355]	250–860 [475]	20–75 [40]	385–510 [435]	600–1915 [1410]
subsp. *pilosum*	1	15	1720	85	3625	10	35	9875	1710	175	1085	2110
**Roots**												
subsp. *tuberculatum*	1	5515	165	25	220	10	60	1400	335	10	145	415
subsp. *sumatranum*	2	8.5–15 [12]	1385–3325 [2356]	20–100 [60]	1510–4185 [2847]	9.5–9.9 [9.6]	30–70 [50]	1025–3615 [2320]	140–320 [230]	5.5–20 [15]	151–264 [208]	537–1431 [984]
subsp. *pilosum*	1	10	770	15	1555	10	15	2825	135	35	175	535
**Stem**												
subsp. *sumatranum*	3	7.6–15 [10]	510–2785 [1470]	6.8–10 [8.3]	720–1335 [1095]	8.4–10 [9.4]	8.5–10 [9.8]	2016.5–3615 [3045]	165–220 [195]	7.1–20 [15]	295–520 [415]	730–1160 [915]
subsp. *pilosum*	1	15	455	5	595	10	10	1210	110	15	155	235
**Bark/phloem**												
subsp. *sumatranum*	1	10	8465	175	11625	10	250	14700	580	115	200	1865
subsp. *pilosum*	1	10	2120	400	9110	5.0	205	6305	680	380	285	1405
**Wood**												
subsp. *sumatranum*	1	5.0	1895	5.0	1335	10	20	1375	130	5.0	140	575
subsp. *pilosum*	1	20	505	5.0	920	10	5.0	1675	130	40	130	545
**Unripe fruit**												
subsp. *sumatranum*	1	45	1955	10	9485	35	25	8075	1445	180	1040	4925
**Mature fruit**												
subsp. *sumatranum*	1	15	2785	10	4525	10	15	13745	1625	130	810	2930

The digest and extracts were analysed with Inductively Coupled Plasma Atomic Emission Spectroscopy (ICP-AES).

### Bio-ore characterisation of *Dichapetalum gelonioides* plants

The production of ash from the dried biomass of the various subspecies of *Dichapetalum gelonioides* resulted in mass reduction factors of about 10–12-fold for *D*. subsp. *tuberculatum* (leaf fraction), *D*. subsp. *pilosum* (twig and leaf fractions) and *D*. subsp. *sumatranum* (leaf fraction), and 50-fold for *D*. subsp. *sumatranum* (stem fraction), with minimal loss of the contained mineral elements. Table 2 gives the elemental concentrations in the dried biomass and the corresponding ash of *D*. subsp. *tuberculatum* (leaf fraction), *D*. subsp. *pilosum* (twig and leaf fractions) and *D*. subsp. *sumatranum* (separate fractions of leaf and stem). Notably, the Ni concentrations in the dried biomass of *D*. subsp. *tuberculatum* (leaf fraction) are exceptionally high, reaching up to 30 265 µg g^−1^. In addition, an extremely high Zn concentration of 10 730 µg g^−1^ is recorded in the dried biomass of *D*. subsp. *sumatranum* (leaf fraction). Regarding the composition of the ‘bio-ore’, as high as 11.3 wt% Ni is recorded in the ash of *D*. subsp. *tuberculatum*, whereas that in the ash of the various plant fractions of *D*. subsp. *pilosum* and *D*. subsp. *sumatranum* are lower than 1.50 wt% Ni (Table 2). In contrast, very high Zn concentrations (1.00–3.00 wt%) are recorded in the ash of the leaf fractions of *D*. subsp. *pilosum* and *D*. subsp. *sumatranum*, and even higher concentrations (3.27 wt%) are found in the stem fraction of *D*. subsp. *sumatranum*, relative to the low Zn concentrations (mean value of 0.25 wt%) in *D*. subsp. *tuberculatum* ash. The concentrations of Ca are significantly high in the ash samples, exceeding 9.50 wt% for all subspecies, and even reaching up to 19.1 wt% in *D*. subsp. *sumatranum ash*. Meanwhile, the K and S concentrations in the ash of *D*. subsp. *pilosum* and *D*. subsp. *sumatranum* (K 8.13–11.8 wt%; S 3.89–7.22 wt%) far exceed that in *D*. subsp. *tuberculatum* (K 2.17–2.28 wt%; S 2.57–2.92 wt%). However, the Mg concentrations in *D*. subsp. *tuberculatum* ash (2.07–2.48 wt%) exceed that in the *D*. subsp. *pilosum* and *D*. subsp. *sumatranum* (0.99–1.85 wt%) ash, and finally, relatively moderate P concentrations (0.55–1.44 wt%) are recorded in the ash of all the subspecies. In contrast to the high concentrations of major elements in the ash of all the subspecies, the concentrations of the trace elements Co, Fe and Mn are relatively low (<0.30 wt%) in the ash samples.

**Table 2:** Elemental concentrations in the dried biomass and the corresponding ash of *Dichapetalum gelonioides* subsp. *tuberculatum*, subsp. *sumatranum* and subsp. *pilosum* (values are given in ranges and means).

*Dichapetalum gelonioides* subsp.	n	Ni	Zn	Al	Ca	Co	Fe	K	Mg	Mn	P	S
		**Elemental concentrations in the dried biomass prior to ashing (µg g** ^**−1**^ **)**										
subsp. *tuberculatum* (Leaves)	2	29535–30265 [29900]	255–275 [265]	40–45 [45]	13525–15845 [14685]	9.3–9.5 [9.4]	175–220 [200]	1335––1400 [1365]	1845–2175 [2005]	40–45 [40]	3490–3885 [3685]	3585–3940 [3760]
subsp. *sumatranum* (Leaves)	1	35	10730	55	19285	3.4	100	12190	2460	190	3290	11435
subsp. *sumatranum* (Stems)	1	5.00	940	45	5530	—	45	2315	265	50	2675	1240
subsp. *pilosum* (Twigs and leaves)	1	15	2995	45	19650	0.5	115	14665	1365	495	3010	9395
		**Elemental concentrations in the ‘bio-ore’ (biomass after ashing) (wt%)**										
subsp. *tuberculatum* (Leaves)	2	10.0–11.0 [10.5]	0.18–0.32 [0.25]	0.05–0.13 [0.09]	9.49–10.1 [9.77]	[0.01]	0.09–0.23 [0.16]	2.17–2.28 [2.23]	2.07–2.48 [2.28]	[0.05]	0.60–0.61 [0.61]	2.55–2.90 [2.75]
subsp. *sumatranum* (Leaves)	1	1.30	2.88	0.13	13.5	—	0.12	8.65	1.85	0.22	0.72	7.22
subsp. *sumatranum* (Stems)	1	0.03	3.27	0.39	19.1	—	0.14	8.13	0.99	0.17	1.14	3.89
subsp. *pilosum* (Twigs and leaves)	1	1.14	1.80	0.07	14.3	—	0.07	11.8	1.14	0.29	0.55	5.80

The digest and extracts were analysed with Inductively Coupled Plasma Atomic Emission Spectroscopy (ICP-AES).

### Light microscopy of woody stems of *Dichapetalum gelonioides*

**Figure 2:**
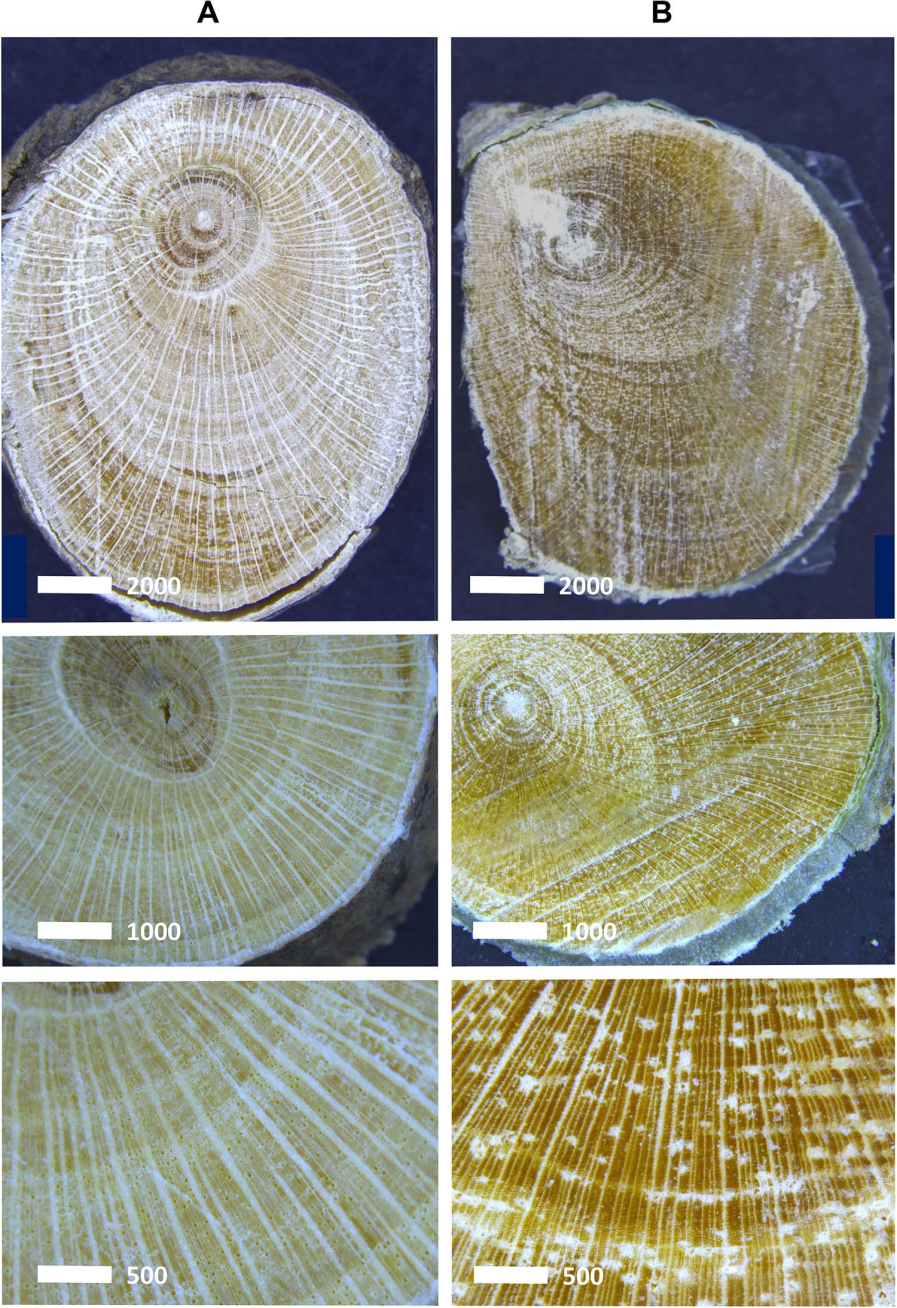
Light electron microscopy of woody stem cross section of (**A**) *D*. subsp. *pilosum* and (**B**) *D*. subsp. *tuberculatum*.

Light microscopy images (Fig. 2) show a whitish phloem in the woody stem of *D*. subsp. *pilosum* whilst in *D*. subsp. *tuberculatum* the phloem is greenish, mainly from the high concentrations of Zn and Ni ions respectively. The images show that Ca crystals are more abundant in the medullary rays of the stem of *D*. subsp. *pilosum* than in *D*. subsp. *tuberculatum* (Fig. 2).

### Synchrotron X-ray fluorescence microscopy investigation

**Figure 3:**
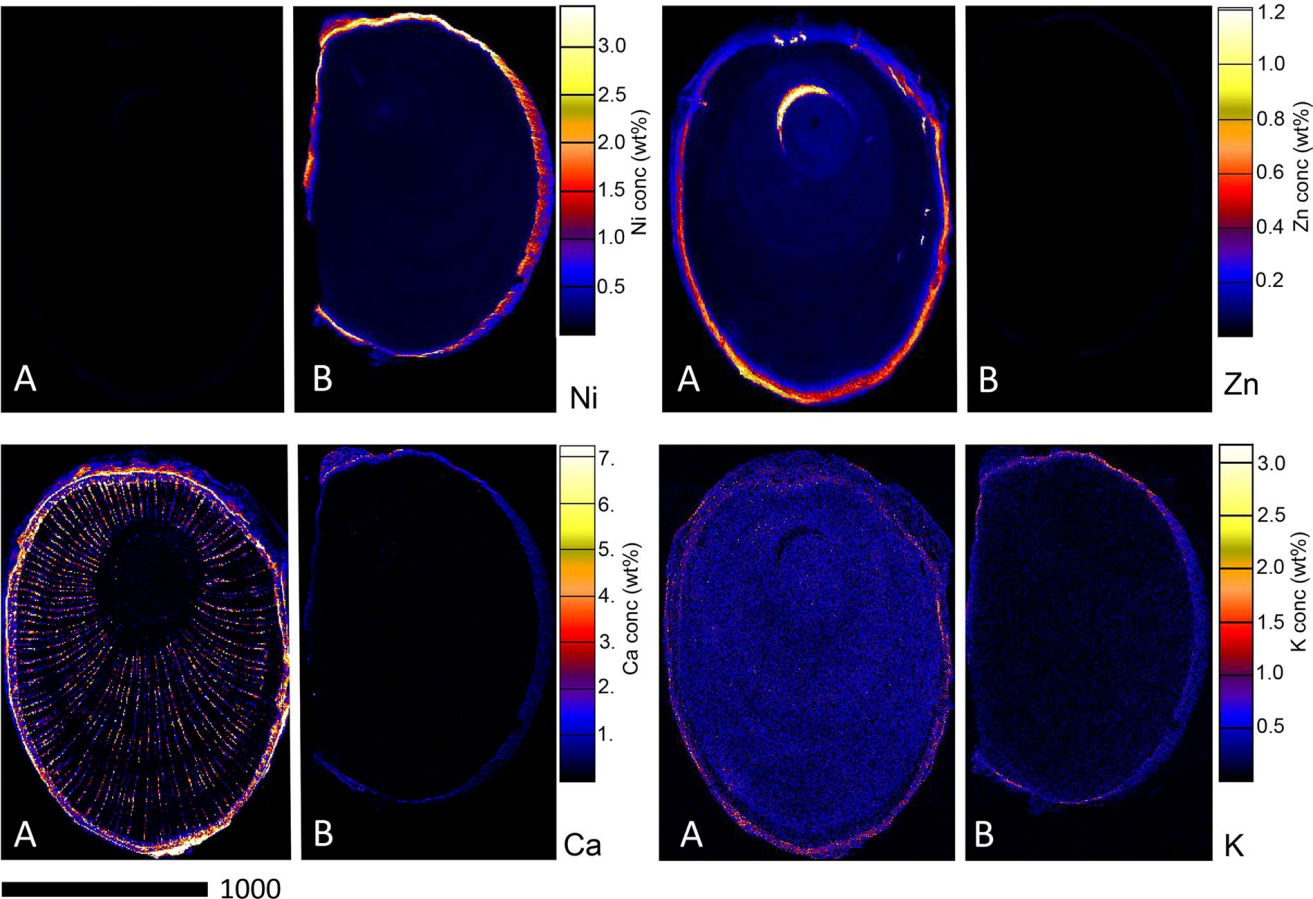
Individual elemental µXRF maps of *Dichapetalum gelonioides* wood sections showing Ni, Zn, Ca, and K distribution in (**A**) *D*. subsp. *pilosum*, and (**B**) *D*. subsp. *tuberculatum*.

Figure 3 shows the elemental distribution in the stems of *D*. subsp. *tuberculatum* and *D*. subsp. *pilosum*. In *D*. subsp. *tuberculatum*, Ni is strongly enriched in the cortex where it reaches up to 3.00 wt%. Moreover, high concentrations of Ni occur in the phloem and epidermis where it reaches up to 1.00 wt%; there is also minor enrichment in the xylem and pith. However, Ni is notably absent throughout the stem of *D*. subsp. *pilosum*. Contrary, Zn is distributed throughout the stem of *D*. subsp. *pilosum*, with strong enrichment in the cortex, with up to 1.20 wt%, followed by the phloem and epidermis where it reaches up to 0.40 wt%. The xylem and pith only show minor enrichment in these elements. In the case of *D*. subsp. *tuberculatum*, Zn is low throughout the stem except in the cortex where there are slightly higher concentrations. Calcium is highly concentrated in the cortex and the epidermis, as well as in the medullary rays of the stem of *D*. subsp. *pilosum*. In *D*. subsp. *tuberculatum*, Ca is only enriched in the cortex, but at concentrations much lower than in *D*. subsp. *pilosum*. Potassium is distributed throughout the stems of both *D*. subsp. *tuberculatum* and *D*. subsp. *pilosum*, but more enriched in the latter than the former and more concentrated in the epidermis.

**Figure 4:**
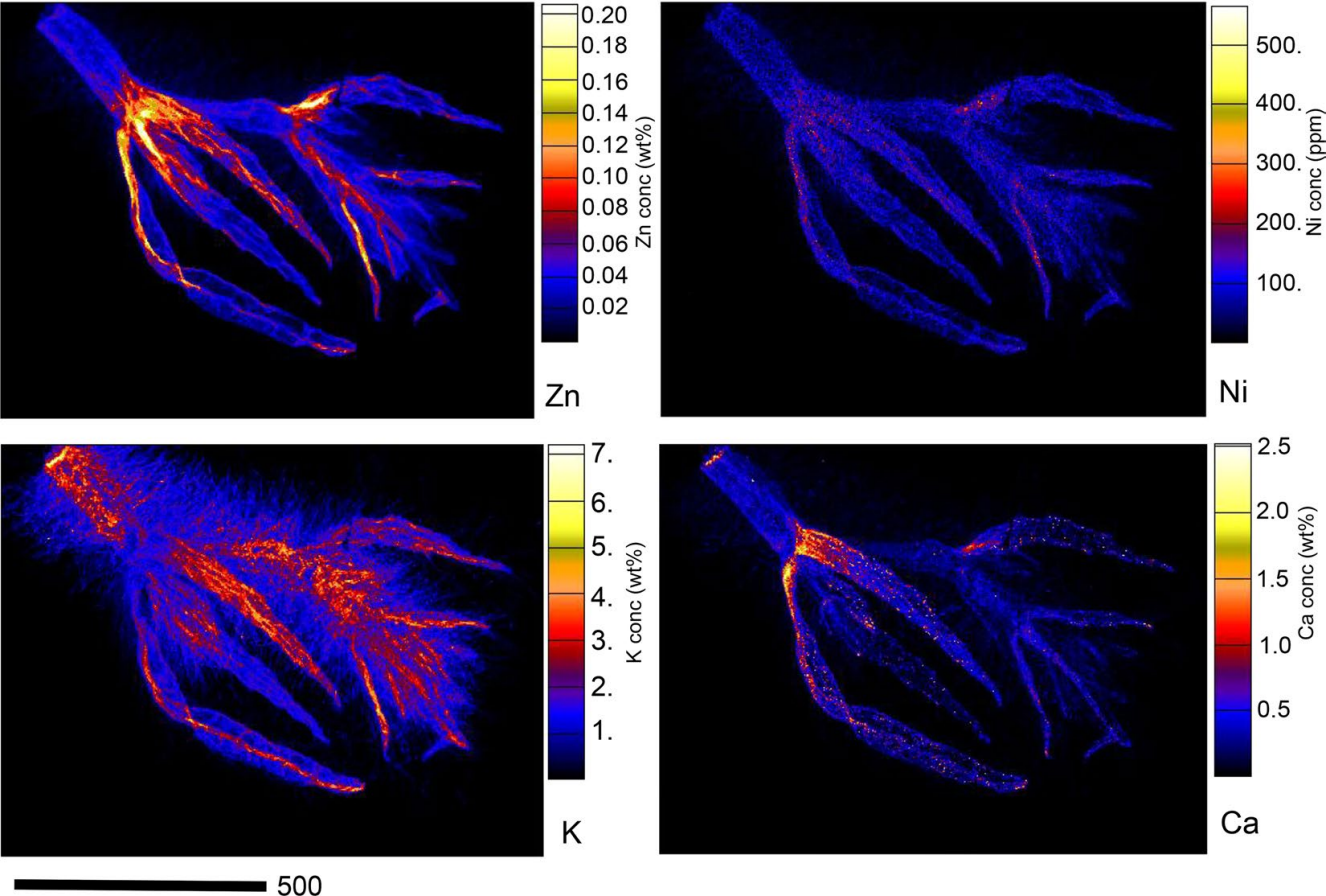
Individual elemental µXRF maps of freeze-dried *D*. subsp. *pilosum* growth bud showing Zn, Ni, K, and Ca distribution.

In the growth bud of *D*. subsp. *pilosum* (Fig. 4), Zn is strongly enriched in the node and the emerging leaves where it reaches up to 0.20 wt%, whereas there is a minor enrichment in the stem with up to 0.08 wt%. Interestingly, the distribution of Ni mirrors that of Zn, but occurs at much low concentrations (<300 µg g^−1^). Potassium is strongly enriched in the stem and the young leaves with minor enrichment in the node, whereas Ca is strongly enriched in the node but has relatively low concentration in the stem and in the emerging leaves.

**Figure 5:**
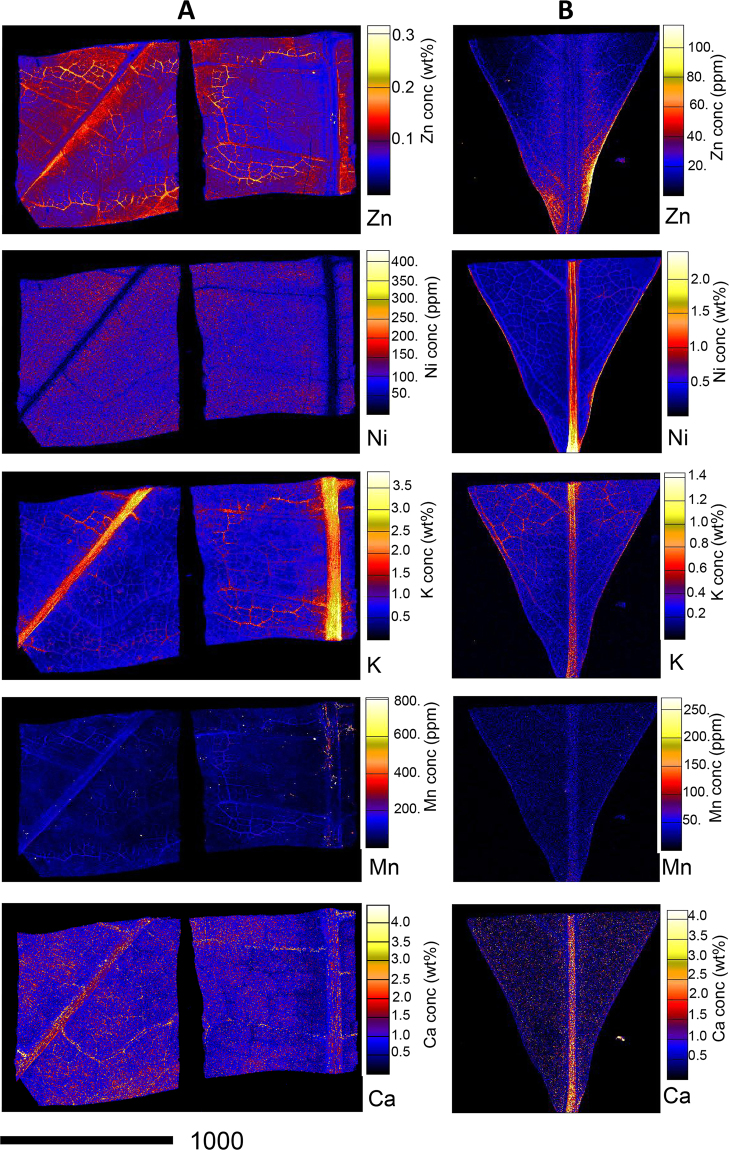
Individual elemental µXRF maps of freeze-dried *Dichapetalum gelonioides* leaves showing Zn, Ni, K, Mn, and Ca distribution in (**A**) *D*. subsp. *pilosum*, and (**B**) *D*. subsp. *tuberculatum*.

Zinc is distributed throughout the leaf of *D*. subsp. *pilosum* with concentrations reaching up to 0.30 wt%, whereas it is distributed at lower concentration in *D*. subsp. *tuberculatum* with concentrations below 100 µg g^−1^ (Fig. 5). Nickel is particularly low in the phloem of the veins of *D*. subsp. *pilosum*. In *D*. subsp. *tuberculatum* Ni is distributed at low concentrations throughout the leaf, but strongly enriched in the phloem of the mid-vein where it exceeds 2.00 wt%. Potassium is strongly enriched in the phloem of the mid-vein of both *D*. subsp. *pilosum* and *D*. subsp. *tuberculatum*. Manganese is distributed more or less uniformly in the leaf of both *D*. subsp. *pilosum* and *D*. subsp. *tuberculatum* at very low concentrations. The distribution of Ca mirrors that of K, albeit with Ca-oxakate deposits lining the veins.

### Rhizosphere soil chemistry of *Dichapetalum gelonioides*

The rhizosphere soil of *D. gelonioides* subsp. *tuberculatum* is near neutral (pH 6.01) as expected for well-buffered ultramafic soils rich in Mg, whereas that of the *D*. subsp. *pilosum* and *D*. subsp. *sumatranum* is acidic (pH 5.05 and 4.64, respectively), typical for leached tropical soils (Table 3). The total Ni concentration is high in the ultramafic soil on which *D*. subsp. *tuberculatum* occurs, unlike that of *D*. subsp. *pilosum* and *D*. subsp. *sumatranum* growing on sandstone soils, where low total soil Ni concentrations (8.50 and 3.50 µg g^−1^, respectively) are recorded. Similarly, the phytoavailable Ni concentrations, as extracted by diethylenetriaminepentaacetic acid (DTPA) and Sr(NO_3_)_2_ solutions, are relatively high in the rhizosphere soil of *D*. subsp. *tuberculatum* (185 µg g^−1^ and 15 µg g^−1^, respectively) compared to that of *D*. subsp. *pilosum* (1.50 µg g^−1^ and 0.30 µg g^−1^, respectively) and *D*. subsp. *sumatranum* (0.70 µg g^−1^ and 0.40 µg g^−1^, respectively). The soil of *D*. subsp. *tuberculatum* also has a higher Mg:Ca ratio and concentrations of Cr, Co, Mn and Fe compared to those of *D*. subsp. *pilosum* and *D*. subsp. *sumatranum*. However, a low concentration of K is recorded in the soil of *D*. subsp. *tuberculatum*, but in the soils of *D*. subsp. *pilosum* and *D*. subsp. *sumatranum* K concentrations are higher. The phytoavailable P concentrations in all the soils are low (<5.00 µg g^−1^). In addition, low concentrations of Zn are recorded for all the rhizosphere soils (total Zn concentrations in the rhizosphere soils of *D*. subsp. *tuberculatum*, *D*. subsp. *pilosum* and *D*. subsp. *sumatranum* are 40, 30 and 20 µg g^−1^, respectively).

**Table 3:** Rhizosphere soil chemistry in the natural habitat of *Dichapetalum gelonioides* subsp. *tuberculatum*, subsp. *sumatranum* and subsp. *pilosum*. Elemental concentrations in µg g^−1^.

*Dichapetalum gelonioides* subsp.	n	pH	Ni	Zn	Mg	P	K	Ca	Cr	Mn	Fe	Co
**Total**												
*tuberculatum*	1	6.01	785	35	28395	105	30	200	1035	1350	23915	50
*sumatranum*	1	4.64	3.6	20	715	85	405	150	3.0	115	4490	1.5
*pilosum*	1	5.05	8.4	35	1285	220	850	685	6.0	335	10680	1.0
**DTPA-extractable**												
*tuberculatum*	1		185	4.6	—	—	—	—	0.17	375	125	10
*sumatranum*	1		0.7	10	—	—	—	—	0.03	60	155	0.5
*pilosum*	1		1.6	6.7	—	—	—	—	0.03	240	160	1.0
**Sr(NO**_**3**_ **)**_**2**_ **-extractable**												
*tuberculatum*	1		10	0.2	—	—	—	—	0.07	6.5	0.5	0.3
*sumatranum*	1		0.4	7.2	—	—	—	—	0.02	60	1.7	0.5
*pilosum*	1		0.3	1.4	—	—	—	—	0.01	110	0.8	0.5

## Discussion

This study confirms that *D*. subsp. *tuberculatum* is a ‘hypernickelophore’ *i.e*., it accumulates >1wt% Ni in the leaves. The rhizosphere soil chemistry implies that *D*. subsp. *tuberculatum* occurs on ultramafic soils. *Dichapetalum gelonioides* subsp. *tuberculatum* is common on Mount Silam (which is part of the Silam-Beeston ultramafic range), and has been recorded only from a few other localities in Sabah^46,50^. The present study thereby confirms the discovery by Baker *et al*.^46^ that *D*. subsp. *tuberculatum* hyperaccumulates Ni when growing on ultramafic soils with leaf Ni concentrations of >25 000 µg g^−1^, showing that *D*. subsp. *tuberculatum* is a strong Ni hyperaccumulator. Datta *et al*.^47^ reported that *D*. subsp. *andamanicum* is also a strong Ni hyperaccumulator with up to 30 000 µg g^−1^ Ni when occurring on ultramafic soils. *Dichapetalum* subsp. *pilosum* and *D*. subsp. *sumatranum* occur on sedimentary (sandstone) bedrock^50^, and have leaf Ni concentrations below 25 µg g^−1^ and are hence not Ni hyperaccumulators. The extraordinary Ni concentrations in the leaves of *D*. subsp. *tuberculatum* (reaching up to 30 265 µg g^−1^) may be exploited for agromining operations. The ‘bio-ore’ (*i.e.* the biomass after ashing) of *D*. subsp. *tuberculatum* contained as high as 11.3 wt% Ni, which is significantly higher than in traditional lateritic ore (which typically has <1 wt% Ni). Similar high-grade ash compositions are recorded in the two most promising species of tropical Ni agromining, *Phyllanthus rufuschaneyi* (12.7 wt%) and *Rinorea cf. bengalensis* (5.50 wt% Ni)^51^. The unique ‘bio-ore’ composition, coupled with the intrinsically high Ni grade, make it possible to extract even higher value products such as Ni catalysts for the organic chemistry industry^52^ and electrochemical Ni products^53^, although smelting of Ni metal is in itself technically feasible^6^. We must add that despite the high purity of the ‘bio-ore’ relative to conventional ore, significant concentrations of Ca (9.49–10.1 wt%), Mg (2.07–2.48 wt%), K (2.17–2.28 wt%) and S (2.57–2.92 wt %) recorded in *D*. subsp. *tuberculatum* are major drawbacks in extracting pure Ni products from this ‘bio-ore’. In other hyperaccumulator ‘metal crop’ species, even higher concentrations of Ca (20–40 wt%), Mg (1.00–5.00 wt%) and K (7.00–11.0 wt%) are recorded in the ‘bio-ore’^51,54^.

**Figure 6:**
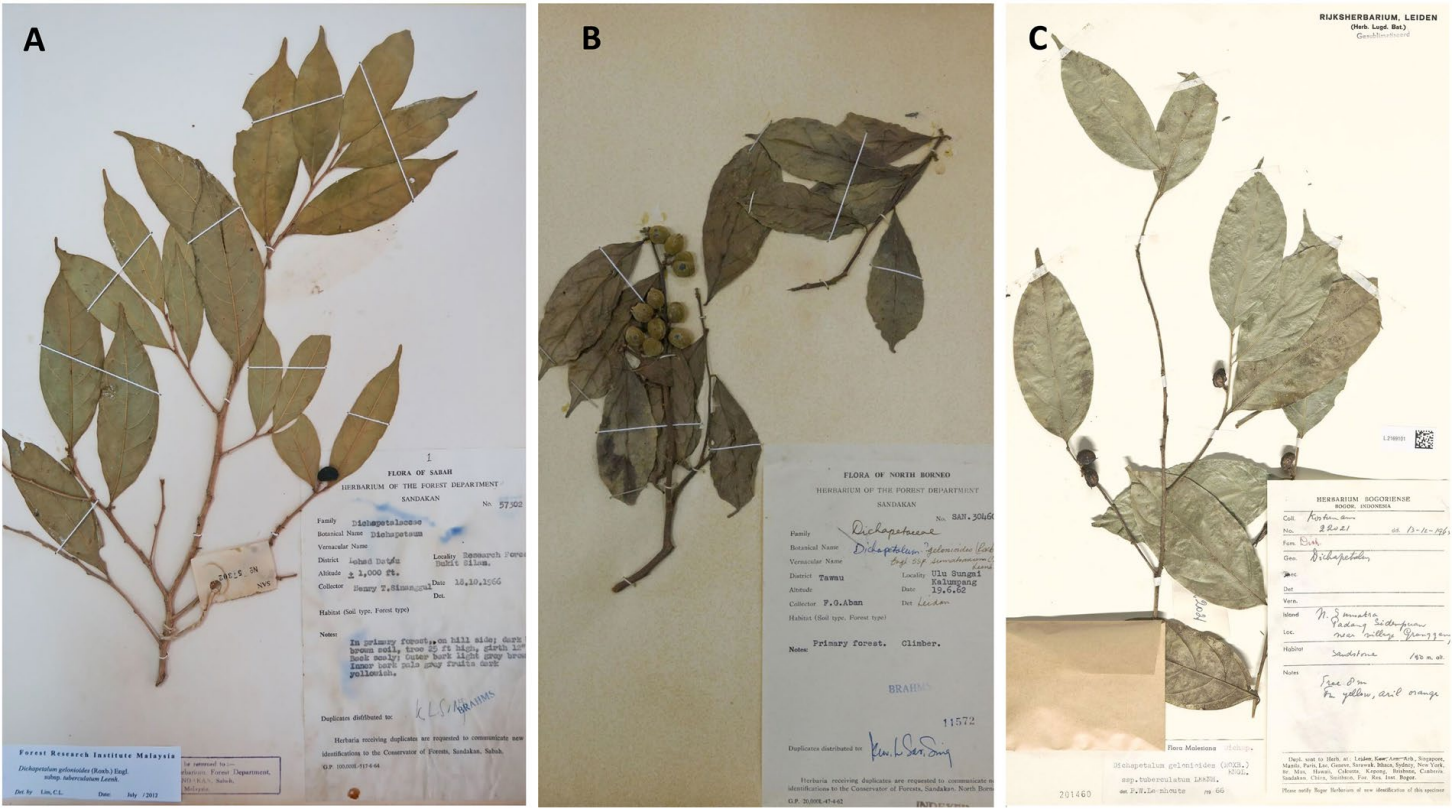
Herbarium specimens of: (**A**) *Dichapetalum gelanioides* subsp*. tuberculatum* (SAN 57302) with 26 650 μg g^−1^ Ni and 257 μg g^−1^ Zn, (**B**) *Dichapetalum gelanioides* subsp. *sumatranum* (SAN 30460) with <5 μg g^−1^ Ni and 14 060 μg g^−1^ Zn, and (**C**) *D. gelonioides* subsp. *tuberculatum* specimen from Sumatra (which had 30 000 µg g^−1^ foliar Zn) (Baker *et al*.^46^) (all images are by A. van der Ent).

The present study reports on the tropical Zn hyperaccumulators, *Dichapetalum* subsp. *sumatranum* and subsp. *pilosum*. Zinc hyperaccumulators are rare globally, with only 15 taxa identified to date^27^. Almost all known Zn hyperaccumulators are herbaceous plants, and the present study is the first to report on a woody Zn hyperaccumulator from field collected material. Most of the known Zn hyperaccumulators occur on Zn enriched (metalliferous) soils. For instance, exceptionally leaf Zn concentrations (53 450 µg g^−1^) have been recorded in a population of *N. caerulescens* growing on soils with extremely high Zn concentrations of up to 64 360 µg g^−1^ in Saint Félix-de-Pallières, France^41^. Similarly high values (53 900 µg g^−1^) have been reported in *A. halleri* from Germany growing on non-metalliferous soils^44^. Zinc hyperaccumulation occuring on soils with ‘normal’ Zn concentrations is known only from these two European species (*A. halleri* and *N. caerulescens*). Reeves *et al*.^41^ analysed field collected specimens of *N. caerulescens* and the corresponding ‘normal’ soils in France and Luxembourg, and found remarkable foliar Zn concentrations of 3230–8890 µg g^−1^ occurring on soils with Zn concentrations of only 115–274 µg g^−1^. Bert *et al*.^29^ also reported 10 876 ± 578 µg g^−1^ Zn concentrations in the aerial part of wild *A. halleri* plants in Germany occurring on soils with 201 ± 30 µg g^−1^ Zn concentrations. The total Zn concentrations in ‘normal’ soils in Sabah are typically between 1.20 to 150 µg g^−1^ ^55^. The present study reveals a remarkable foliar Zn concentration of 10 730 µg g^−1^ in *D*. subsp. *sumatranum* growing on ‘normal’ soils with total Zn concentration of only 20 µg g^−1^. It is noteworthy that the concentrations we report are not the highest for this species, but that Baker *et al*.^46^ found as high as 14 000 µg g^−1^ and 25 000 µg g^−1^ in herbarium specimen of *D*. subsp. *sumatranum* and *D*. subsp. *pilosum* respectively (Fig. 6), showing that *Dichapetalum* species are among the strongest Zn hyperaccumulators globally. Regarding the (Zn) Bioaccumulation Coefficients (BC) reported for Zn hyperaccumulator species, the values for species occurring on metalliferous soils are relatively lower than that of the non-metalliferous soils^41,44^. Previously, the highest BC reported for a Zn hyperaccumulator species was recorded in *A. halleri* occurring on non-metalliferous soils with a BC of ~1270^44^. The present study also reveals an exceptional BC of ~500 for *D*. subsp. *sumatranum*, further confirming the extraordinary accumulation behaviour of *Dichapetalum* species. We discovered that *Dichapetalum gelonioides* is the only (Zn) hyperaccumulator known to occur exclusively on normal soils, while hyperaccumulating Zn.

In *Dichapetalum*, Zn accumulation at extreme concentrations is not only limited to the leaves, but in other plant tissues as well, even though the distribution is uneven. The present study reveals as high as 8465 µg g^−1^ Zn concentrations in the phloem/bark of *D*. subsp. *sumatranum*. This exceptionally high Zn concentration in the phloem may be transported to the fruits and seeds. The mature fruit of *D*. subsp. *sumatranum* has high Zn concentrations of up to 2785 µg g^−1^. The high Zn concentration in the phloem and the fruits of *D*. subsp. *sumatranum* could have implications for Zn biofortification of edible crop plants. For crop plants, Zn concentrations decrease in the order of roots >shoots >fruits and seeds^56^. Notably, low Zn mobility in phloem limits the Zn concentrations in fruits and seeds, and these phloem-fed tissues rarely achieve Zn concentrations greater than 30–100 µg g^−1 ^^15^. As a result, Zn concentrations are very low in grain, seed, fruit or tuber crops^57–59^. Consequently, Zn deficiency disorders are more prevalent in population with diets dominated by such crops^60^. Zinc is an essential trace element for all organisms, and particularly important for human nutrition^57,58,61^. The Institute of Medicine USA^62^ recommends a daily intake of 8–13 mg Zn, but up to 1/3 of the global population has inadequate dietary Zn intake because of limited access to Zn-rich foods, and consequently, Zn deficiency is a major health problem in developing countries^63–66^. Particularly, it is more problematic in Southeast Asia because of the consumption of diets low in Zn, mainly polished rice. Limited dietary Zn intake can be minimised by improving the available Zn in the diet, and biofortification of edible crops is a sustainable strategy especially in developing countries^15,67–69^. The target Zn concentrations are 28 µg g^−1^ in rice, and 38 µg g^−1^ in wheat grain and maize^68^. Zinc hyperaccumulators have the potential to advance biofortification, because insights into the biomolecular mechanisms of Zn acquisition may be applied to food crops^15^, and hyperaccumulator plant biomass may be used as a dietary supplement^14^. However, *A. halleri* and *N. caerulescens* are both temperate region plants with low biomass, and hence unsuited for cultivation in tropical regions where human Zn deficiency is prevalent. Hence, the discovery of a Zn hyperaccumulator plant species on ‘normal’ soils in Sabah provides a strong incentive to advance Zn biofortification measures in Southeast Asia. Insights gained from the mechanisms underlying the remarkable Zn translocation and accumulation especially in the phloem and phloem-fed tissues may be particulary useful. Evidence from transcriptome data suggests that Zn hyperaccumulator plant species have an enhanced capacity to protect cells from metal toxicity^22,27^. On the other hand, crop plants maintain low Zn concentration in the phloem sap as a strategy to avoid cellular toxicity^58^. Therefore, identifying the genes involved in the Zn hyperaccumulation in *D*. subsp. *sumatranum* could enable the breeding of genetically modified edible crops with enhanced abilities, especially translocation of Zn in the phloem. The increased Zn tolerance and mobility in phloem of these improved cultivars would therefore enrich the phloem-fed tissues (such as seeds/grains), and ultimately increase dietary Zn intake in Southeast Asia.

Another approach to increase dietary Zn intake in Southeast Asia may be to introduce the biomass of hyperaccumulators, such as *D*. subsp. *sumatranum* and *D*. subsp. *pilosum,* as a food supplement^14^. Considering a recommended intake of 8 mg Zn (Institute of Medicine USA^62^), an average 33% efficiency of human uptake^70^, and a leaf Zn concentration of 9000 µg g^−1^ in the leaves of *D*. subsp. *sumatranum* (Table 2), consuming 0.5 g dry material would meet one fifth of the daily requirements. As Zn in the leaves of Zn hyperaccumulators is likely bioavailable, human Zn uptake from these materials should not be an issue. However, there are major food safety concerns regarding the use of Zn hyperaccumulator plant species as food because *A. halleri* and *N. caerulescens* can also accumulate Cd in their leaves^41^. Cadmium is highly toxic to humans, and obviously consuming plant tissues with high bioavailable Cd is not recommended for human health^71^. Therefore, there is a need to test whether *Dichapetalum*-species occurring on ‘normal’ soils will accumulate Cd. Furthermore, many *Dichapetalum*-species are extremely toxic to livestock because they contain fluorinated compounds such as fluoroacetic acid and ω-fluorinated fatty acids^72–76^. Therefore, these fluorinated compounds will need to be detoxified before they are introduced as food supplements for human consumption, and toxicological assays are clearly required. Some *Dichapetalum*-species can also contain fluorine-free compounds, such as the triterpenoids group of *dichapetalins* that are being intensively investigated because of their strong cytotoxic activity, and potential application in anti-cancer drugs^77–81^.

Apart from the potential for agromining and biofortification applications, *Dichapetalum*-species can advance our understanding on both Ni and Zn hyperaccumulation. The mechanisms involved in the hyperaccumulation of trace elements are not fully understood, but the key processes include uptake by roots and xylem loading as well as sequestration in the leaf cells. Whether hyperaccumulation is primarily driven by root processes, or hypertolerance is determined by shoot processes remains inconclusive, at least in tropical hyperaccumulator plant species. Grafting experiments in which the rootstocks and shoot scions are reciprocally grafted from *D*. subsp. *pilosum* or *D*. subsp. *sumatranum* (Zn hyperaccumulating subsp.) and *D*. subsp. *tuberculatum* (Ni hyperaccumulating subsp.) may unravel the distinct roles of root and shoot processes in hyperaccumulation and hypertolerance. In addition, reciprocal dosing of *Dichapetalum*-species in which the Zn hyperaccumulating subsp. is dosed with Ni, and *vice versa* may provide more insights into the uptake, translocation, tolerance and accumulation of these trace elements. Furthermore, Ni and Zn stable isotope tracing experiments can provide useful information on both uptake and transfer mechanisms within the plant-soil systems. Therefore, *Dichapetalum*-species are an attractive model to study both Ni and Zn hyperaccumulation and hypertolerance in tropical hyperaccumulator plant species.

## Materials and Methods

### Herbarium XRF scanning

The Niton XL3t 980 analyser (Thermo-Fisher Scientific) uses a miniaturised X-ray tube (Ag anode; 6–50 kV, 0–200 µA max) as its excitation source. The X-ray tubes irradiates the sample with a stable source of high-energy X-rays, and fluorescent X-rays are detected, identified and quantified by the inbuilt Silicon Drift Detector (SDD) with ~185 eV, up to 60 000 cps, 4 µs shaping time. The instrument uses Compton normalisation for quantification, appropriate for the relatively low elemental concentrations found in plant material. A total of 590 dried plant samples originating from Sabah, Malaysia^82^ were used for the calibration. From each sample, a 6-mm diameter leaf disc (to match the XRF beam width) was extracted using a paper punch. A square of ~99.7% pure titanium (2 mm thick × 10 cm × 10 cm; Sigma-Aldrich 369489–90G) was used behind the specimens to provide a uniform background and block penetrating X-rays. XRF measurements were carried out in the ‘Soils Mode’ for 60 s. After scanning, the leaf samples were weighed, digested and analysed with Inductively Coupled Plasma Atomic Emission Spectroscopy (ICP-AES), following the procedures described below. Correction factors were derived by linear regression of XRF data against corresponding ICP-AES measurements.

In total, 91 herbarium species were measured at the herbarium of the Forest Research Centre in Sepilok, Sabah, Malaysia. This comprised two species: *Dichapetalum grandifolium* and *Dichapetalum gelonioides* (subsp. *tuberculatum, pilosum, sumatranum*). Each specimen was measured for 30 seconds in ‘Soil Mode’ and the raw XRF data was corrected using the regression formulas obtained from the calibration.

### Collection and analysis of plant tissue and rhizosphere soil samples

**Figure 7:**
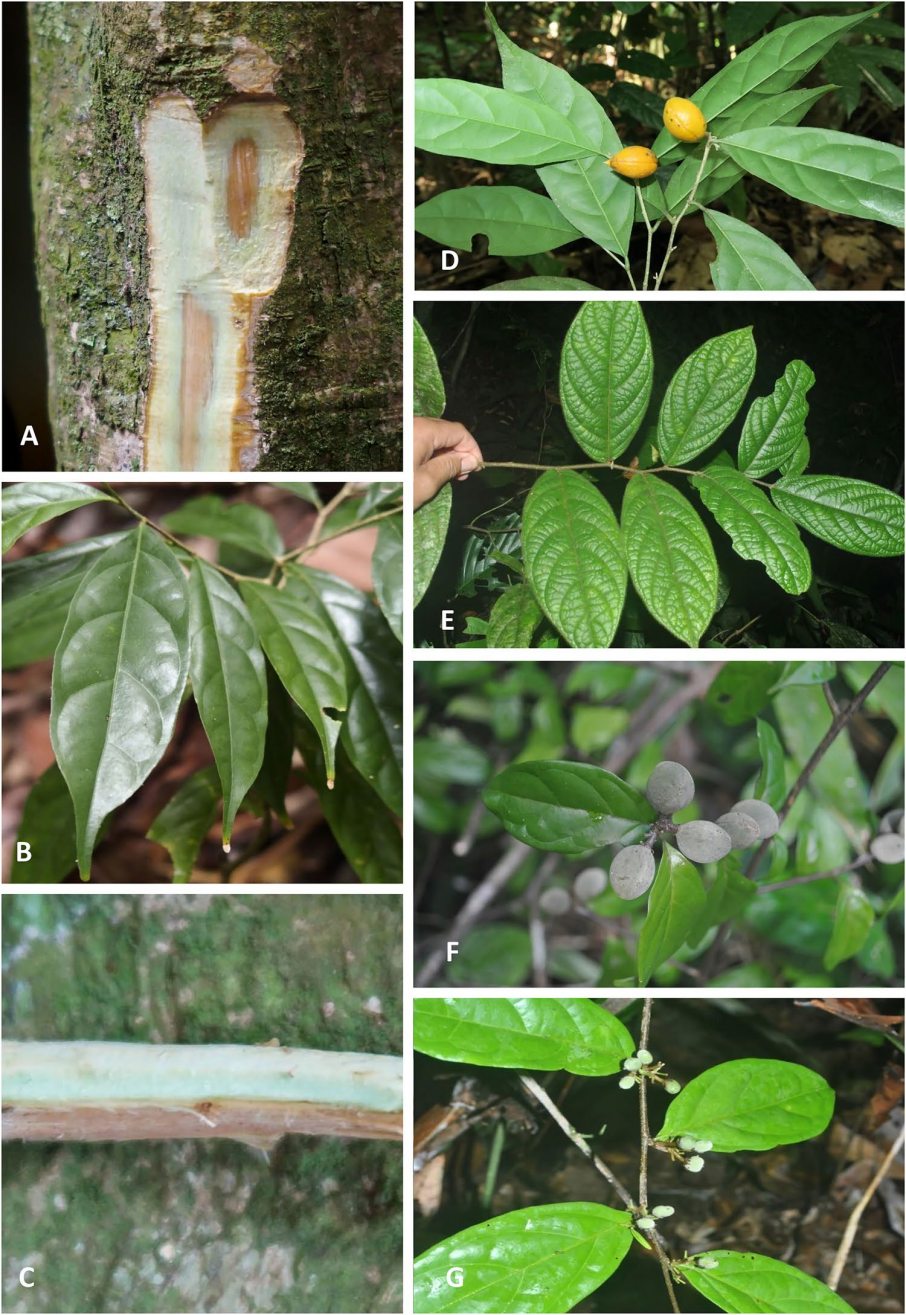
The various *D. gelonioides* subspecies in the native habitat in Sabah: *D*. subsp. *tuberculatum* (**A**–**D**), *D*. subsp. *pilosum* (**E**) and *D*. subsp. *sumatranum* (**F**,**G**) (photos by Antony van der Ent and Sukaibin Sumail).

Plant tissue samples (leaves, wood, bark, flowers) for bulk chemical analysis were collected in the natural habitats in Sabah, Malaysia: *D. gelonioides* subsp. *tuberculatum* (Mt Silam Forest Reserve), and *D*. subsp. *pilosum* and *D*. subsp. *sumatranum* (Sepilok-Kabili Forest Reserve) (Fig. 7). These samples were dried at 70 °C for five days in a drying oven and subsequently packed for transport to Australia and gamma irradiated at Steritech Pty. Ltd. in Brisbane following Australian Quarantine Regulations. The dried plant tissue samples were subsequently ground and ~300 mg material digested using 4 mL HNO_3_ (70%) and 1 mL H_2_O_2_ (30%) in a microwave oven (Milestone Start D) for a 45-minute programme and diluted to 30 mL with ultrapure water (Millipore 18.2 MΩ·cm at 25 °C). Further, bulk samples of *D. gelonioides* subsp. *tuberculatum* (leaves), D. subsp. *sumatranum* (leaves), *D*. subsp. *sumatranum* (stems), and *D*. subsp. *pilosum* (twigs and leaves) were collected. After oven-drying at 70 °C, subsamples of ~750 mg were ashed in a muffle furnace at 550 °C for 5 hrs and cooled to room temperature. Samples were weighed before and after ashing for gravimetric analysis. The ash was subsequently dissolved in 37% HCl (10 mL per sample), and analysed with ICP-AES as described below.

Rhizosphere soil samples were collected from near the roots of the *Dichapetalum* plants. Soil sub-samples (~300 mg) were digested using 9 mL 70% HNO_3_ and 3 mL 37% HCl per sample in a digestion microwave (Milestone Start D) for a program of 1.5 hours, and diluted to 45 mL with ultrapure water before analysis to obtain pseudo-total elemental concentrations. Soil pH was obtained in a 1 to 2.5 soil to water mixture after 2 hr shaking. Exchangeable trace elements were extracted in 0.1MSr(NO_3_)_2_ at a soil:solution ratio of 1:4 (10 gram soil with 40 mL solution) and 2 hr shaking time (adapted from Kukier & Chaney^83^). As a means of estimating potentially phytoavailable trace elements, the DTPA-extractant was used according to Becquer *et al*.^84^, which was adapted from the original method by Lindsay and Norvell^85^, with the following modifications: excluding triethanolamine (TEA), adjusted at pH 5.3, 5 g soil with 25 mL extractant, and extraction time of one hr. The plant digests and soil digests/extracts were analysed with ICP-AES (Varian Vista Pro II) for Ni, Co, Cr, Cu, Zn, Mn, Fe, Mg, Ca, Na, K, S and P; the Certified Reference Materials (CRMs) are given in Supplementary Table 2. Because of the low concentrations of our target elements in the Standard Reference Material Apple Leaves NIST 1515, we have included other CRMs (Standard Reference Material Tomato Leaves NIST 1573a and Standard Reference Material Spinach Leaves NIST 1570a) with relatively high Ni and Zn concentrations for improved accuracy (see Supplementary Table 2).

### Collection and preparation of samples for Synchrotron X-ray Fluorescence Microscopy (XFM)

Plant tissue samples (roots, wood, leaves and phloem tissue) and rhizosphere soil samples were collected in the native rainforest habitat in Malaysian Borneo (Sepilok-Kabili Forest Reserve for *D*. subsp*. sumatranum* and *D*. subsp*. pilosum* and Mount Silam Forest Reserve for *D*. subsp. *tuberculatum*, in Sabah, Malaysia). Tissue samples intended for synchrotron analysis were excised with a razor blade, and immediately shock-frozen using a metal mirror technique in which the samples were pressed between a block of Cu-metal cooled by liquid N_2_ and a second Cu-metal block attached to a Teflon holder. This ensured extremely fast freezing of the plant tissue samples to prevent cellular damage by ice crystal formation. The samples were then wrapped in Al-foil, and transported in a cryogenic container (kept at <180 °C).

The samples required freeze-drying because they could not be kept frozen during the X-ray fluorescence microscopy (XFM) analysis. The samples were freeze-dried (using a Thermoline freeze-dryer) employing a long process of 4 days in which the temperature was stepwise increased, starting at −196 °C on a liquid N_2_ pre-cooled block (with the freeze-dryer set to −85 °C) until room temperature, to limit morphological changes of the tissues and elemental re-distribution.

### Synchrotron X-ray Fluorescence Microscopy

The X-ray fluorescence microscopy (XFM) beamline employs an in-vacuum undulator to produce a brilliant X-ray beam. An Si(111) monochromator and a pair of Kirkpatrick-Baez mirrors delivers a monochromatic focused incident beam onto the specimen^86^. The Maia detector uses a large detector array to maximize detected signal and count-rates for efficient imaging. Maia enables high overall count-rates and uses an annular detector geometry, where the beam passes though the detector and strikes the sample at normal incidence^87,88^. This enables a large solid-angle (1.2 steradian) to be achieved in order to either maximize detected signal or to reduce the dose and potential damage to a specimen^89^. Maia is designed for event-mode data acquisition, where each detected X-ray event is recorded, tagged by detector number in the array, position in the scan and other metadata^90^. This approach eliminates readout delays and enables arbitrarily short pixel times (typically down to 0.1 ms) and large pixel count to be achieved for high definition imaging (typically 10–100 M pixels). The freeze-dried samples were mapped by sampling at intervals ranging from 2–10 μm and 0.5–5 ms transit time. The XRF event stream was analysed using the Dynamic Analysis method^91,92^ as implemented in GeoPIXE^93^.

## Electronic supplementary material

Supplementary Information
