# Supplementary material for: Contrasting nickel and zinc hyperaccumulation in subspecies of Dichapetalum gelonioides from Southeast Asia
Source: Sci Rep. 2018 Jun 25;8:9659. doi: 10.1038/s41598-018-26859-7 (PMC6018115; doi:10.1038/s41598-018-26859-7)
Supplement: Supplementary file 1 — Supplementary Information [file 41598_2018_26859_MOESM1_ESM.pdf]

Contrasting nickel and zinc hyperaccumulation in subspecies of *Dichapetalum gelonioides* from Southeast Asia

Philip Nti Nkrumah[1], Guillaume Echevarria[2], Peter D. Erskine[1] and Antony van der Ent[1,2*]

[1]Centre for Mined Land Rehabilitation, Sustainable Minerals Institute, The University of Queensland, Queensland, Australia.

[2]Université de Lorraine-INRA, Laboratoire Sols et Environnement, UMR 1120, France.

*corresponding author: a.vanderent@uq.edu.au

Supplementary Table 1. X-ray Fluorescence Spectroscopy (XRF) concentration data (mg kg^{-1}) of herbarium specimens in the genus *Dichapetalum* held at the Forest Research Centre Herbarium in Sepilok, Sabah, Malaysia. Abbreviations: <LOD is lower than limit of detection for XRF.

Sample ID	Mn	Co	Ni	Zn	Genus	Species	Subspecies	Date	Locality	Gazetteer
36007	85	<LOD	31705	400	*Dichapetalum*	*gelonioides*	*tuberculatum*	27/05/1963	Lahad Datu	Silam
33353	140	<LOD	25930	265	*Dichapetalum*	*gelonioides*	*tuberculatum*	4/03/1963	Lahad Datu	Lahad Datu
36019	80	<LOD	24065	1150	*Dichapetalum*	*gelonioides*	*tuberculatum*	28/05/1963	Lahad Datu	Silam
57302	<LOD	<LOD	23595	285	*Dichapetalum*	*gelonioides*	*tuberculatum*	18/10/1966	Lahad Datu	Silam
57282	135	<LOD	17410	240	*Dichapetalum*	*gelonioides*	*tuberculatum*	16/10/1966	Lahad Datu	Silam
57302	<LOD	<LOD	14000	975	*Dichapetalum*	*gelonioides*	*tuberculatum*	18/10/1966	Lahad Datu	Silam
SAN21684	120	<LOD	13210	635	*Dichapetalum*	*gelonioides*	*tuberculatum*	27/03/1961	Lahad Datu	Ulu Segama FR
SAN68326	<LOD	<LOD	11775	85	*Dichapetalum*	*gelonioides*	*tuberculatum*	15/05/1970	Lahad Datu	Mostyn
46145	140	<LOD	10560	1370	*Dichapetalum*	*gelonioides*	*tuberculatum*	22/10/1965	Lahad Datu	Mostyn
SAN96938	<LOD	<LOD	9710	220	*Dichapetalum*	*gelonioides*	*tuberculatum*	7/06/1983	Beluran	Telupid
SAN117015	110	<LOD	8345	175	*Dichapetalum*	*gelonioides*	*tuberculatum*	24/01/1987	Beluran	Ulu Tungud FR
SAN108912	<LOD	<LOD	6275	365	*Dichapetalum*	*gelonioides*	*tuberculatum*	8/07/1985	Lahad Datu	Ulu Segama FR
SAN108694	100	<LOD	6140	110	*Dichapetalum*	*gelonioides*	*tuberculatum*	29/03/1985	Lahad Datu	Ulu Segama FR
36358	150	65	4570	1415	*Dichapetalum*	*gelonioides*	*tuberculatum*	9/07/1963	Kinabatangan	Segaliud Lokan FR
36392	95	<LOD	3460	1335	*Dichapetalum*	*gelonioides*	*tuberculatum*	12/07/1964	Kinabatangan	Segaliud Lokan FR
SAN107849	<LOD	<LOD	2760	35	*Dichapetalum*	*gelonioides*	*tuberculatum*	10/03/1985	Lahad Datu	Ulu Segama FR
SAN108913	<LOD	<LOD	2695	510	*Dichapetalum*	*gelonioides*	*tuberculatum*	9/07/1985	Lahad Datu	Ulu Segama FR
36389	75	<LOD	790	2665	*Dichapetalum*	*gelonioides*	*tuberculatum*	11/07/1963	Kinabatangan	Segaliud Lokan FR
SAN74588	435	<LOD	105	915	*Dichapetalum*	*gelonioides*	*pilosum*	10/12/1971	Beluran	Beluran
SAN50532	1645	<LOD	60	4985	*Dichapetalum*	*gelonioides*	*pilosum*	12/10/1966	Tenom	Tomani
SAN28375	2890	<LOD	<LOD	10610	*Dichapetalum*	*gelonioides*	*pilosum*	21/11/1961	Sandakan	SepilokFR
SAN71291	335	<LOD	<LOD	10505	*Dichapetalum*	*gelonioides*	*pilosum*	23/11/1970	Beluran	Segaliud Lokan FR
SAN31896	1305	<LOD	<LOD	7280	*Dichapetalum*	*gelonioides*	*pilosum*	17/11/1962	Tenom	Sapong
39637	1365	<LOD	<LOD	6910	*Dichapetalum*	*gelonioides*	*pilosum*	2/10/1963	Sandakan	Kebun Cina

Supplementary Table 1. Cont.

Sample ID	Mn	Co	Ni	Zn	Genus	Species	Subspecies	Date	Locality	Gazetteer
SAN139861	430	<LOD	<LOD	5915	*Dichapetalum*	*gelonioides*	*pilosum*	21/11/1994	Sandakan	Sandakan
SAN27736	1130	<LOD	<LOD	5870	*Dichapetalum*	*gelonioides*	*pilosum*	10/10/1961	Sandakan	Sibuga
SAN64733	2010	<LOD	<LOD	5550	*Dichapetalum*	*gelonioides*	*pilosum*	8/05/1969	Sandakan	Sepilok FR
43605	1955	<LOD	<LOD	4645	*Dichapetalum*	*gelonioides*	*pilosum*	5/05/1964	Sandakan	Sepilok FR
SAN91827	<LOD	<LOD	<LOD	4000	*Dichapetalum*	*gelonioides*	*tuberculatum*	24/04/1980	Tawau	Kalabakan FR
49206	730	<LOD	<LOD	3890	*Dichapetalum*	*gelonioides*	*pilosum*	4/08/1965	Ranau	Ranau
SAN128789	715	<LOD	<LOD	3860	*Dichapetalum*	*gelonioides*	*pilosum*	15/05/1990	Sandakan	Sepilok FR
SAN141427	2335	<LOD	<LOD	3550	*Dichapetalum*	*gelonioides*	*pilosum*	7/10/1994	Sandakan	Kebun Cina
SAN80879	345	<LOD	<LOD	3455	*Dichapetalum*	*gelonioides*	*tuberculatum*	17/06/1976	Nabawan	Pensiangan
SAN95130	280	<LOD	<LOD	3320	*Dichapetalum*	*gelonioides*	-	30/03/1982	Kinabatangan	Tongod
SAN101721	1055	<LOD	<LOD	3295	*Dichapetalum*	*gelonioides*	*tuberculatum*	21/09/1983	Kinabatangan	Tongod
SAN90292	1210	<LOD	<LOD	3125	*Dichapetalum*	*gelonioides*	*pilosum*	21/05/1982	Sandakan	Sepilok FR
SAN85661	290	<LOD	<LOD	2785	*Dichapetalum*	*gelonioides*	*tuberculatum*	20/05/1977	Keningau	Crocker Range
SAN132887	100	<LOD	<LOD	2755	*Dichapetalum*	*gelonioides*	*tuberculatum*	19/07/1991	Sipitang	Maligan FR
SAN83679	255	<LOD	<LOD	2715	*Dichapetalum*	*gelonioides*	-	15/04/1977	Sandakan	Sepilok FR
SAN79675	<LOD	<LOD	<LOD	2595	*Dichapetalum*	*gelonioides*	*tuberculatum*	8/07/1974	Tawau	Tawau Hill FR
SAN110948	1595	<LOD	<LOD	2520	*Dichapetalum*	*gelonioides*	*pilosum*	19/03/1985	Keningau	Pingas Pingas
SAN96594	<LOD	<LOD	<LOD	2480	*Dichapetalum*	*gelonioides*	*pilosum*	13/04/1983	Ranau	Ranau
54543	<LOD	<LOD	<LOD	2335	*Dichapetalum*	*gelonioides*	*tuberculatum*	15/04/1966	Lahad Datu	Mostyn
SAN117913	1345	<LOD	<LOD	2210	*Dichapetalum*	*gelonioides*	*pilosum*	12/06/1987	Ranau	Bambangan
SAN139563	655	<LOD	<LOD	2000	*Dichapetalum*	*gelonioides*	*pilosum*	25/08/1994	Nabawan	Pensiangan
SAN67455	145	<LOD	<LOD	1950	*Dichapetalum*	*gelonioides*	*sumatranum*	5/02/1985	Kinabatangan	Tongod
SAN119109	510	<LOD	<LOD	1950	*Dichapetalum*	*gelonioides*	*pilosum*	22/09/1987	Sandakan	Kebun China
SAN100189	155	<LOD	<LOD	1915	*Dichapetalum*	*gelonioides*	*tuberculatum*	12/09/1983	Kinabatangan	Tongod
SAN118040	1215	<LOD	<LOD	1910	*Dichapetalum*	*gelonioides*	*pilosum*	24/01/1989	Sandakan	Sepilok FR
SAN111165	700	<LOD	<LOD	1850	*Dichapetalum*	*gelonioides*	*pilosum*	17/10/1985	Tawau	Tawau Hill Park

Supplementary Table 1. Cont.

Sample ID	Mn	Co	Ni	Zn	Genus	Species	Subspecies	Date	Locality	Gazetteer
SAN78183	<LOD	<LOD	<LOD	1835	*Dichapetalum*	*gelonioides*	*pilosum*	18/06/1974	Beaufort	Bukau
SAN144517	200	<LOD	<LOD	1820	*Dichapetalum*	*gelonioides*	*pilosum*	6/02/2003	Sandakan	Sepilok FR
41188	745	<LOD	<LOD	1795	*Dichapetalum*	*gelonioides*	*pilosum*	6/02/1964	Kota Belud	Kelawat Forest Reserve
SAN136788	285	<LOD	<LOD	1780	*Dichapetalum*	*gelonioides*	*pilosum*	18/01/1994	Tenom	Mandalom Forest Reserve
SAN83319	270	<LOD	<LOD	1675	*Dichapetalum*	*gelonioides*	*tuberculatum*	20/05/1976	Kinabatangan	Lamag
SAN87350	435	<LOD	<LOD	1580	*Dichapetalum*	*gelonioides*	*pilosum*	19/08/1977	Tawau	Luasong
SAN78479	1095	<LOD	<LOD	1550	*Dichapetalum*	*gelonioides*	*pilosum*	7/03/1975	Beaufort	Gn. Lumaku FR
SAN76238	650	<LOD	<LOD	1500	*Dichapetalum*	*gelonioides*	*pilosum*	25/09/1972	Kota Belud	Kota Belud
SAN94824	150	<LOD	<LOD	1495	*Dichapetalum*	*gelonioides*	*tuberculatum*	25/05/1982	Tawau	Kalabakan FR
SAN95708	125	<LOD	<LOD	1480	*Dichapetalum*	*gelonioides*	*pilosum*	14/03/1983	Tawau	Kalabakan FR
SAN116329	320	<LOD	<LOD	1400	*Dichapetalum*	*gelonioides*	*pilosum*	9/10/1986	Ranau	Mamut
SAN30146	170	<LOD	<LOD	1345	*Dichapetalum*	*gelonioides*	*pilosum*	27/05/1962	Tawau	Balung
SAN30688	<LOD	<LOD	<LOD	1305	*Dichapetalum*	*grandifolium*	-	23/06/1962	Sandakan	Sandakan
SAN90834	600	<LOD	<LOD	1235	*Dichapetalum*	*gelonioides*	*pilosum*	9/06/1979	Tenom	Tomani
SAN81046	2485	<LOD	<LOD	1175	*Dichapetalum*	*gelonioides*	*pilosum*	12/12/1974	Ranau	Tampias
SAN129925	295	<LOD	<LOD	1130	*Dichapetalum*	*gelonioides*	*pilosum*	15/05/1992	Nabawan	Milian Labau FR
SAN118515	1555	<LOD	<LOD	1060	*Dichapetalum*	*gelonioides*	*pilosum*	27/10/1986	Keningau	Lanas
SAN124097	365	<LOD	<LOD	1055	*Dichapetalum*	*gelonioides*	*pilosum*	17/06/1988	Tenom	Mandalom
SAN135212	<LOD	<LOD	<LOD	920	*Dichapetalum*	*gelonioides*	*pilosum*	9/10/1996	Tawau	Kalumpang FR
39937	85	<LOD	<LOD	905	*Dichapetalum*	*grandifolium*	-	14/10/1963	Lahad Datu	Silabukan FR
SAN25961	<LOD	<LOD	<LOD	795	*Dichapetalum*	*grandifolium*	-	25/09/1961	Lahad Datu	Kalumpang FR

Supplementary Table 1. Cont.

Sample ID	Mn	Co	Ni	Zn	Genus	Species	Subspecies	Date	Locality	Gazetteer
SAN109934	400	<LOD	<LOD	785	*Dichapetalum*	*gelonioides*	*pilosum*	17/06/1985	Keningau	Keningau
SAN143503	590	<LOD	<LOD	700	*Dichapetalum*	*gelonioides*	*pilosum*	23/06/2000	Kunak	Madai Baturong FR
53053	0	<LOD	<LOD	645	*Dichapetalum*	*grandifolium*	-	8/02/1966	Lahad Datu	Lahad Datu
SAN115873	195	<LOD	<LOD	600	*Dichapetalum*	*gelonioides*	*tuberculatum*	28/05/1986	Nabawan	Nabawan
54898	<LOD	<LOD	<LOD	570	*Dichapetalum*	*grandifolium*	-	14/07/1966	Lahad Datu	Lahad Datu
SAN100282	135	<LOD	<LOD	445	*Dichapetalum*	*gelonioides*	*pilosum*	27/08/1984	Kinabatangan	Sg. Pinangah FR
SAN135742	485	<LOD	<LOD	440	*Dichapetalum*	*gelonioides*	*pilosum*	22/07/1993	Kinabatangan	Penangah FR
39999	75	<LOD	<LOD	395	*Dichapetalum*	*grandifolium*	--	10/11/1963	Lahad Datu	Diwata
SAN114479	425	<LOD	<LOD	380	*Dichapetalum*	*gelonioides*	*pilosum*	1/07/1986	Nabawan	Sapulut
SAN20446	<LOD	<LOD	<LOD	350	*Dichapetalum*	*grandifolium*		18/11/1959	Lahad Datu	Mostyn
SAN127666	210	<LOD	<LOD	265	*Dichapetalum*	*gelonioides*	*pilosum*	23/06/1989	Nabawan	Bkt. Pisagan
SAN124509	0	<LOD	<LOD	250	*Dichapetalum*	*grandifolium*	-	13/10/1988	Beluran	Segaliud Lokan
40243	1065	<LOD	<LOD	165	*Dichapetalum*	*gelonioides*	*pilosum*	22/06/1964	Beaufort	Beaufort
SAN79139	<LOD	<LOD	<LOD	125	*Dichapetalum*	*grandifolium*	-	18/08/1975	Tawau	Tawau
SAN95551	<LOD	<LOD	<LOD	90	*Dichapetalum*	*grandifolium*	-	10/12/1982	Tawau	Kalabakan FR
SAN124254	<LOD	<LOD	<LOD	45	*Dichapetalum*	*grandifolium*		19/07/1988	Beluran	Telupid
SAN135148	450	<LOD	<LOD	10	*Dichapetalum*	*gelonioides*	*pilosum*	23/06/1996	Kinabatangan	Tangkulap VJR

Supplementary Table 2a. Certified Reference Materials (Apple Leaves NIST 1515) (values are given in µg g^{-1}).

Replicate	Ca	Co	Cr	Fe	K	Mg	Mn	Ni	P	Zn
1	15099	1.1	1.4	92	15997	2834	52	3.1	1399	20
2	15339	0.7	1.1	83	16182	2901	58	3.7	1485	20
3	15110	1.4	1.9	83	16026	2793	56	4.1	1506	18
4	15303	0.5	0.8	87	15990	2937	51	3.2	1573	19
5	15248	0.5	1.2	79	16231	2984	53	2.2	1434	16
6	13288	0.2	0.3	50	14326	2390	45	1.2	1431	12
7	13741	0.2	2.7	56	15135	2565	47	0.6	1463	17
8	15012	6.4	0.9	73	15844	2667	56	1.5	1541	14
9	14930	5.5	2.2	73	15771	2648	53	1.4	1586	13
mean	**14785**	**1.8**	**1.3**	**75**	**15722**	**2746**	**52**	**2.3**	**1490**	**16.5**
stdev	**245**	**0.7**	**0.2**	**4.5**	**204**	**64**	**1.4**	**0.4**	**20**	**1.0**
**Standard Reference Material Apple Leaves NIST 1515**										
Certified values	15260	0.1	0.3	83	1610	2710	54	0.9	1590	13

Notes: the in-house method diverted from the NIST-method as samples were not dehydrated prior to analysis, and digests were not treated with HF to dissolve remaining silicates.

Supplementary Table 2b. Certified Reference Materials (Tomato Leaves NIST 1573a and Spinach Leaves NIST 1570a) (values are given in µg g^{-1}).

Sample	Ca	Cr	Fe	K	Mg	Mn	Ni	P	Zn
NIST 1573a	5038	2.1	356	2719	1135	242	1.6	2114	29
NIST 1570a	1514	2.0	216	2910	863	82	2.5	5185	81
**Standard Reference Material (Certified values)**									
NIST 1573a	5050	2.0	368	2700	1200	246	1.59	2160	31
NIST 1570a	1520	–	–	2900	890	76	2.14	5200	82

NIST 1573a: Standard Reference Material Tomato Leaves

NIST 1570a: Standard Reference Material Spinach Leaves

Notes: the in-house method diverted from the NIST-method as samples were not dehydrated prior to analysis, and digests were not treated with HF to dissolve remaining silicates.
